# GaN Nano Air Channel Diodes: Enabling High Rectification Ratio and Neutron Robust Radiation Operation

**DOI:** 10.1002/advs.202310300

**Published:** 2024-06-27

**Authors:** Yazhou Wei, Feiliang Chen, Yu Zhang, Ruihan Huang, Haiquan Zhao, Mo Li, Jian Zhang

**Affiliations:** ^1^ School of Electronic Science and Engineering University of Electronic Science and Technology of China (UESTC) Chengdu 611731 China; ^2^ Institute of Advanced Millimeter‐Wave Technology University of Electronic Science and Technology of China (UESTC) Chengdu 611731 China; ^3^ Yangtze Delta Region Institute University of Electronic Science and Technology of China (UESTC) Huzhou 313000 China

**Keywords:** high rectification ratio, low‐power consumption, nano air channel, neutron radiation, rectifying nanodiodes

## Abstract

Nano air channel transistors (NACTs) provide numerous advantages over traditional silicon devices, including faster switching speeds, higher operating frequencies, and enhanced radiation hardness attributable to the ballistic transport of electrons. In the development of field‐emission‐based integrated circuits, low‐power consumption rectifying nano air channel diodes (NACDs) play a crucial role. However, achieving rectification characteristics in NACDs is challenging due to their structural and material symmetry. This paper proposes a vertical GaN NACD with a consistent nano air channel fabricated using IC‐compatible processes. The GaN NACD exhibits an exceptionally low turn‐on voltage of 0.3 V while delivering a high output current of 5.02 mA at 3 V. Notably, it demonstrates a high rectification ratio of up to 2.2 × 10^5^, attributing to significant work function disparities within the GaN‐Au structure, coupled with the reduction of Au surface roughness to minimize reverse current. Furthermore, the junction‐free structure and superior material properties of GaN enable the NACD to be suitable for use in radiation‐rich environments. With its potential as a fundamental component of ultrafast and ultrahigh‐frequency integrated circuits, this intriguing and cost‐effective rectifying diode is anticipated to garner widespread interest within the electronics community.

## Graphical abstract

A vertical GaN nano air channel diode (NACD) is prepared by IC‐compatible processes with an exceptionally low turn‐on voltage of 0.3 V while delivering a high output current of 5.02 mA@3 V, as well as a high rectification ratio of up to 2.2 × 10^5^ for ultrafast and ultrahigh‐frequency integrated circuits in radiation environments.

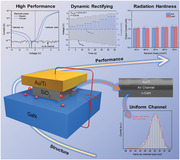

## 1 Introduction

The development of nano air channel transistors (NACTs) has received considerable attention in the past decade.^[^ ^1^, ^2^, ^3^ ^]^ Driven by the advantages of ballistic transport, as well as the scalability, low cost, and reliability achieved through micro/nanomanufacturing techniques, NACTs with air channels smaller than the electron mean free path in air (≈68 nm) can be fabricated.^[^ ^2^, ^4^, ^5^ ^]^ NACTs inherently have superiority over solid‐state devices in terms of ultra‐fast electrical response in the femtosecond level^[^ ^6^, ^7^ ^]^ and a cut‐off frequency in terahertz range due to their unique ability to transport electrons without encountering scattering effects or heat limitations, unlike semiconductors where carriers are susceptible to optical and acoustic phonon scattering.^[^ ^8^ ^]^ Another notable advantage of NACTs is their superior resistance to high temperature and radiation, attributed to their junction‐free nature.^[^ ^9^ ^]^ The on‐chip integration capability of NACTs further facilitates the realization of high‐performance integrated circuits.^[^ ^10^, ^11^ ^]^ As a result, NACTs have emerged as an ideal choice for high‐frequency and high‐speed systems.

In the advancement of NACTs, nano air channel diodes (NACDs) play a crucial role in various applications such as integrated circuits,^[^ ^12^ ^]^ sensors,^[^ ^13^ ^]^ and ultrafast electronics.^[^ ^7^ ^]^ However, previous NACDs have faced challenges in achieving rectification characteristics due to their symmetrical material and structure at both terminals.^[^ ^5^, ^13^, ^14^, ^15^, ^16^, ^17^ ^]^ To overcome this obstacle, researchers have explored different approaches. One approach involves fabricating NACDs with a tip‐edge structure, which creates noticeable differences in electric field strength between the anode and cathode. Planar tip‐edge structures built by electron beam lithography (EBL) or focused ion beam (FIB) techniques have exhibited excellent rectification capabilities and low‐power consumption (<3 V).^[^ ^18^, ^19^, ^20^ ^]^ However, their practical applications are severely constrained by the high cost and limited mass‐production capability of nanofabrication technologies. Another example involves the self‐assembly of polystyrene nanospheres to create metal‐based NACDs with sub‐10 nm air channels and rectification ratios of 10^5^.^[^ ^7^ ^]^ Unfortunately, the inconsistency of process parameters has hampered the advancement of these devices. Another practical approach to rectifying properties is employing materials with different work functions. A vertical structure^[^ ^21^ ^]^ consisting of a Si cathode and an Au anode was reported, exhibiting a rectification ratio of about 10 with a non‐negligible reverse current due to the small work function difference. Zhao et al.^[^ ^22^ ^]^ introduced a planar NACD with AlGaN/GaN as the cathode and Au as the anode, showcasing an exceptional rectification ratio of more than 10^4^ due to the significant difference in work function. Moreover, Siwapon et al.^[^ ^2^, ^6^, ^23^ ^]^ proposed FIB‐prepared vertical metal‐oxide‐semiconductor (Al‐SiO_2_‐Si) structures with nano air channels, which exhibited significant rectification ratios from 10^2^ to 10^4^. In addition to the inherent characteristics of the structural asymmetry and materials with different work functions, the roughness of the electrode plays an essential role in the field emission behavior. Previous studies have reported that increasing the surface roughness of diamond and graphene electrodes can lead to an elevation in the field‐emission current density and a reduction in the turn‐on field.^[^ ^24^, ^25^ ^]^ However, research on modulating roughness to enhance rectification characteristics remains inadequate.

Gallium nitride (GaN) offers a range of outstanding properties that are highly advantageous for NACT applications, including low electron affinity in the range of 2.7–3.3 eV,^[^ ^26^, ^27^ ^]^ radiation hardness, high breakdown field of 3.3 MV cm^−1^,^[^ ^28^, ^29^ ^]^ and superior physical and chemical stability. The substantial difference in work function (1.1 eV) achievable by using the GaN‐Au structure in NACTs promises to deliver notable rectification performance.

In our previous research, we successfully fabricated vertical GaN NACDs using the plasma‐enhanced chemical vapor deposition (PECVD) method, effectively addressing the issue of wafer‐scale fabrication.^[^ ^30^ ^]^ Here, we propose a GaN‐Au vertical NACD featuring a high rectification ratio and a high output current at low‐power consumption. We achieve uniform and consistent nano air channels by employing the atomic layer deposition (ALD) technique with atomic‐level precision control and other IC‐compatible technologies. This device achieves high rectification performance by designing a significant difference in the work function within the GaN‐Au vertical structure and reducing the Au surface roughness to minimize the reverse current. Our GaN NACD exhibits impressive performance characteristics, including an output current of 5.02 mA@3 V, a low turn‐on voltage of 0.3 V, and a high static rectification ratio of 2.2 × 10^5^. Additionally, we investigate the dynamic rectification properties of the proposed NACD under different voltage and frequency conditions, encompassing both square wave and sine signals. Notably, the device's tolerance to fast neutron radiation is evaluated, which is crucial in assessing its potential applications in nuclear applications. Our research findings underscore the promising potential of NACD in future nanoelectronics applications while providing valuable insights that contribute to the design and development of NACDs.

## 2 Results and Discussion

### 2.1 Device Design and Preparation

**Figure 1:**
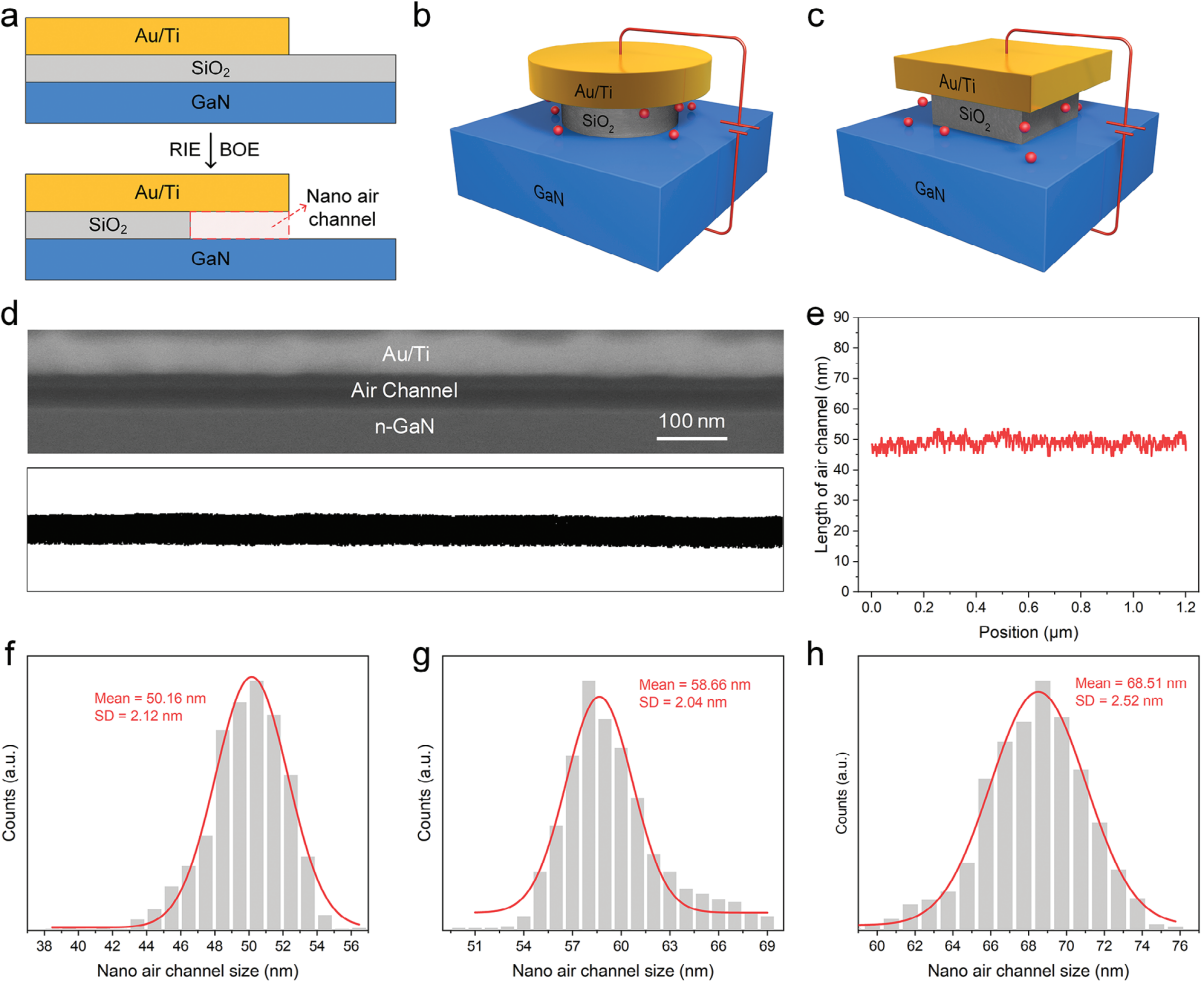
Design and preparation of the GaN NACDs. a) The controllable preparation of nano air channels. The SiO_2_ outside the electrodes and between the electrodes and GaN was removed by RIE and BOE wet etching processes, resulting in the formation of nano air channels with a length consistent with the thickness of the oxide layer. b,c) GaN NACDs with a circular electrode (b) and a square electrode (c), respectively, with identical electrode circumferences. d) SEM image and corresponding binary visualization of the nano air channel formed between the electrode and GaN substrate, where only the areas within the air channel are displayed as dark pixels. e) The relationship between the length and position of the air channel is extracted from the binary image. f,g,h) The histogram summarizes the size distribution of 50 nm (f), 60 nm (g), and 70 nm (h) air channels. Air channel lengths were extracted using the method of Kano et al.^[^ ^35^ ^]^

This paper proposes a vertical NACD consisting of a GaN‐Au configuration with a significant difference in work function. The vertical GaN NACD was fabricated using IC‐compatible processes, including ALD and reactive ion etching (RIE). **Figure** **1**a illustrates the principle of a controllable nano air channel, where the channel length is determined by the thickness of the oxide layer, which can be precisely controlled by adjusting the thickness of the sacrificial layer deposited using ALD. Further details regarding the device preparation process can be found in our previous publication^[^ ^30^ ^]^ and the Experiment Section. The oxide layer prepared by ALD exhibits atomic‐level precision, facilitating precise control of the sacrificial layer thickness and enhancing its uniformity, thereby improving its stability. Additionally, this process reduces the surface roughness^[^ ^31^, ^32^ ^]^ of the Au electrode deposited on the oxide layer. By diminishing the field enhancement factor,^[^ ^33^ ^]^ the reverse field emission current of the Au electrode can be reduced, consequently enhancing the rectification characteristics. Previous studies have elucidated the correlation between field emission current and surface roughness across various electrode materials, including diamond,^[^ ^25^ ^]^ Cu–Cr,^[^ ^34^ ^]^ and graphene electrodes.^[^ ^24^ ^]^ Here, we define that when the Au electrode is biased positively/negatively, the output current from the GaN/Au cathode is designated as the forward/reverse current, while the GaN remains in a zero‐bias state throughout. Scanning electron microscope (SEM) images of the cross‐section of the device before and after buffered‐oxide etchant (BOE) wet etching are shown in Figure S1 (Supporting Information). Additionally, Figure 1b,c illustrate GaN NACDs featuring circular and square electrodes, respectively, having equal circumferences of 8000 µm and etching depths of ≈400 nm. Considering that the field emission area is a product of the electrode perimeter and the etching depth, these configurations possess the same field emission area of 3200 µm^2^.

The performance and stability of NACDs are greatly influenced by the uniformity and consistency of the nano cair channels. The nanogap measurement method proposed by Kano et al.^[^ ^35^ ^]^ was adopted to evaluate the uniformity of the fabricated nano air channels accurately. This method transforms SEM images into binary images that solely depict the nano air channels, as shown in Figure 1d. By analyzing the distribution of dark pixels, which represent the size information of the channels, we can effectively assess their uniformity. The relationship between the positions and lengths of the extracted air channels in the binary image is depicted in Figure 1e.

The histograms shown in Figure 1f–h depict the size distribution of air channels with widths of 50, 60, and 70 nm air channels, respectively. The data were obtained from SEM images of three samples (see Figure S2, Supporting Information). The peaks of the histograms are centered ≈50.16, 58.66, and 68.51 nm, respectively, with standard deviations below 2.60 nm. These results indicate a consistent and uniform fabrication of the nano air channels. Compared to channels fabricated using PECVD, the uniformity of the ALD‐formed air channels is significantly improved, as evidenced by the 4.28 nm reduction in the standard deviation of the length distribution (see Figure S3, Supporting Information). This reduction in the standard deviation contributes to a smoother surface roughness of the Au electrode, enabling a solution for increasing the rectification ratio of the device by reducing the Au emission current. Theoretically, this fabrication method has the potential to achieve a sub‐nanometer distribution standard deviation in channels on atomically flat silicon substrates. However, for air channels fabricated on GaN, the uniformity is primarily influenced by the surface roughness of GaN. The surface roughness of GaN, as observed using an atomic force microscope (AFM), is given in Figure S4 (Supporting Information).

### 2.2 Electrical Characteristics of GaN NACDs

**Figure 2:**
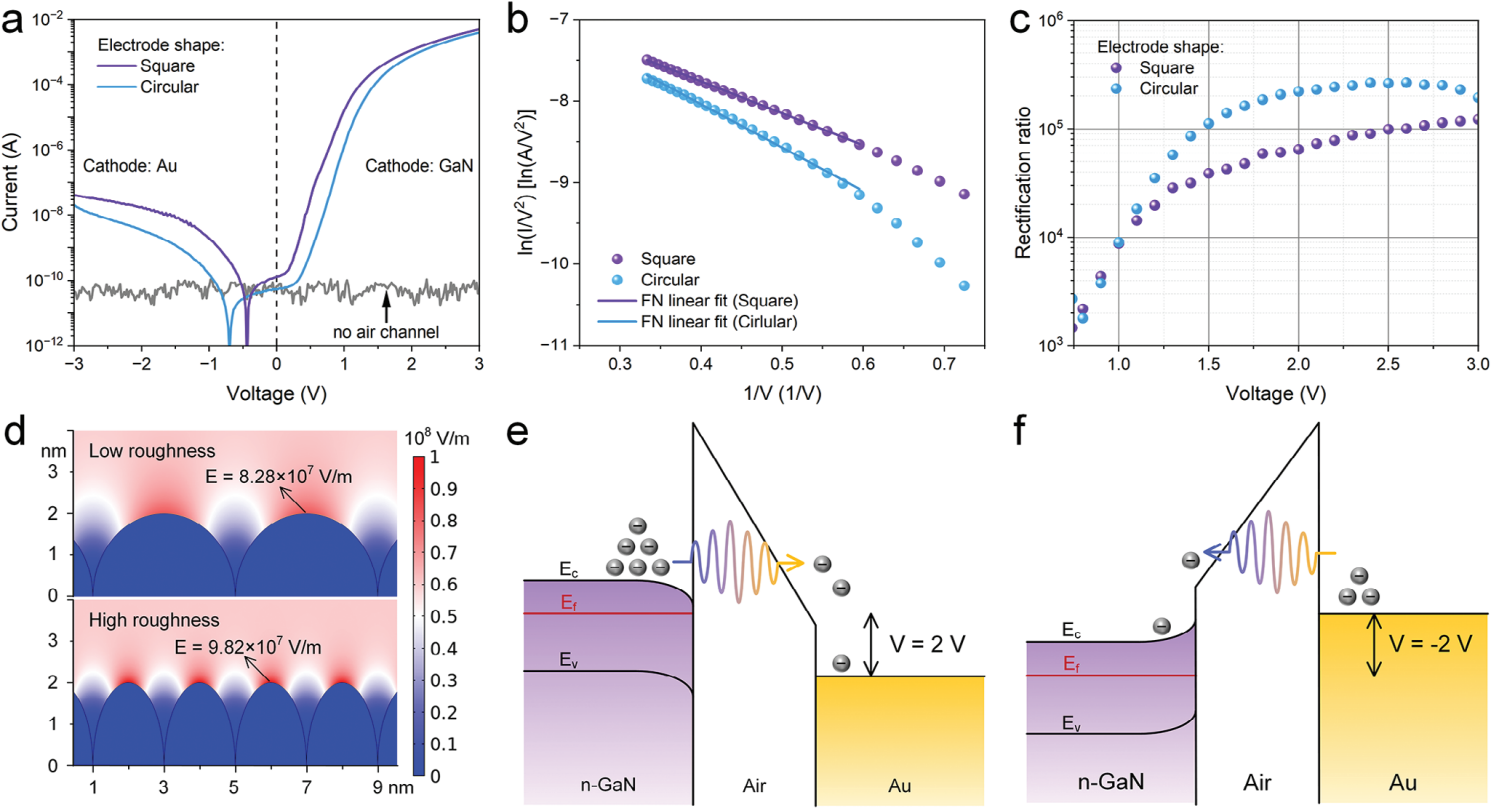
Electrical characteristics of GaN NACDs. a,b,c) *I*–*V* characteristics (a), FN curves (b), and rectification ratio (c) of devices with square and circular electrodes. d) Simulation of electric field distribution on high and low roughness surfaces. e,f) Illustration of the energy band diagram of GaN NACD at positive bias (e) and negative bias (f). E_c_, E_f_, and E_v_ denote the conduction band, Fermi level, and valence band, respectively.

**Figure** **2**a presents the electrical characteristics of both circular and square electrode NACDs. The current–voltage (*I‐* *V*) characteristics exhibit high rectifying characteristics at low‐power consumption, with low currents (less than 10^−7^ A) under reverse bias. Devices without these channels show extremely low currents below 10^−10^ A, confirming the presence of electrically isolating nano air channels. The square electrode device's forward and reverse turn‐on voltages (*V*_ON_s) are 0.3 and −0.88 V, respectively, while those of the circular electrode device are 0.54 and −1.46 V, respectively. The *V*_ON_ is defined as the voltage required to obtain 1 nA^[^ ^36^ ^]^ of emission current. It is evident that the NACD with a square electrode exhibits a higher forward current, with output currents of 5.02 mA at 3 V. The linear relationship between ln(*I*/*V* ^2^) and 1/*V* in Figure 2b indicates that the device follows Fowler‐Nordheim (FN) theory, describing the relationship between the output current and the anode voltage as follows:^[^ ^37^ ^]^

(1) $$\ln\left(I/V^{2}\right)=\ln A-B/V$$

where A = 1.56 × 10^−6^ *αβ* ^2^ *φ* ^−1^ *d* ^−2^ and B = 6.83 × 10^9^ *φ* ^3/2^ *dβ* ^−1^, respectively. Here *α* is the field emission area, *β* is the field enhancement factor, *φ* is the work function of the cathode and *d* is the length of the air channel. The electron emission at <1 V follows the Schottky emission mechanism, as shown in Figure S5 (Supporting Information). The square electrode influences the distribution of the electric field within the air channel, thereby increasing the local electric field on the GaN surface and resulting in a higher *β* (with *β* of 605.68 and 513.20 for square and circular electrodes, respectively), consequently leading to a higher forward current compared to devices with circular electrodes of the same field emission area. The simulation of the effect of electrode geometry on the electric field is shown in Figure S6 (Supporting Information).

The impact of electrode shape also extends to the reverse characteristics. The bias voltage was swept from −3 to 3 V during the evaluation of the device's static characteristics. In the case of the device with the circular electrode, the current level reached zero at ≈−0.7 V, indicating a transition from reverse to forward current polarity. Meanwhile, the presence of non‐zero current at zero bias is attributed to the capacitive effects^[^ ^38^ ^]^ arising from the Au/SiO_2_/GaN capacitor‐like structure. Our device is equivalent to a parallel resistor with a capacitor. To obtain a better understanding of this capacitive behavior, we performed cyclic *I–V* characteristic measurements over a voltage range of −3 V to 3 V, as shown in Figure S7 (Supporting Information). These *I–V* characteristics illustrate the typical charge and discharge cyclic curves for a capacitor with parallel resistance. The NACD with a square electrode exhibits a more significant zero‐current point (−0.44 V) due to the accumulation of a larger electric field at the corner, leading to a higher field enhancement factor and a smaller *V*_ON_. This behavior can be observed by fitting the reverse current in the FN plot, as shown in Figure S8 (Supporting Information). In addition, the performance of devices with different channel lengths and material work functions are discussed in Figures S9 and S10 (Supporting Information), respectively. The breakdown characteristics of the device with a 50 nm air channel are presented in Figure S11 (Supporting Information). To further validate the stability and reproducibility of the GaN NACD, we conducted experiments under air and vacuum conditions, as shown in Figure S12 (Supporting Information).

Figure 2c displays the rectification ratio (*RR*) of the two devices. In this context, *RR* is defined as *RR* = |*I*(*V*_a_)/*I*(−*V*_a_)|, where *I*(*V*_a_) is the output current at an applied voltage *V*_a_. Both devices exhibit *RR* values >10^4^ with an applied voltage >1 V. Nevertheless, there exists a disparity in the trend of the *RR* values between the two devices. The device with circular electrodes demonstrates an increase in the *RR* as the voltage is raised, exceeding 10^5^ at 1.5 V, and stabilizing at 2 V. Conversely, the square electrode device exhibits consistently increasing *RR* values, exceeding 10^5^ at a voltage of 2.5 V. **Table** **1** summarizes the electrical performance of our device in comparison to other proposed NACDs. Our design stands out by incorporating low roughness Au electrodes, which effectively reduces the reverse current. Furthermore, we have achieved high rectification at low voltages through the utilization of IC‐compatible processes.

**Table 1:** Comparison of electrical performance of rectification ratio devices.

Device type	Material [Cathode–Anode]	Fabrication method	Rectification reason	Channel [nm]	*V*_ON_ [V]	Current [µA@V]	Rectification ratio
Planar diode	AlGaN/GaN‐Au^[^ ^22^ ^]^	BOE	Difference in work function	45	2.3	40@3	≈10^4^
	VO_2_(A)‐VO_2_(A)^[^ ^18^ ^]^	FIB	Structural asymmetry	10	0.46	53@1	>50
	Ta–Ta^[^ ^19^ ^]^	EBL	Structural asymmetry	24	∼0.5	0.62@1	48
	Au–Au^[^ ^7^ ^]^	Nanosphere lithography	Structural asymmetry	6.88	0.7	0.987@2	10^6^
	GaN–GaN^[^ ^20^ ^]^	EBL	Structural asymmetry	30	0.24	0.457@1	500
Vertical diode	Si–Au^[^ ^2^ ^]^	FIB	Difference in work function	23	0.5	0.1@1	≈10
	Si–Ga/Graphene^[^ ^6^ ^]^	FIB	Difference in work function	23	‐	1.4@1	≈300
	Si–Ga/Graphene/Al^[^ ^23^ ^]^	FIB	Difference in work function	23	‐	450@1	10^4^
	Si–Au^[^ ^21^ ^]^	BOE	Difference in work function	80	1.08	160@2	16
This work	GaN–Au	BOE	Differences in work function, roughness	50	0.3	1161@2	2.2 × 10^5^

Figure 2c exhibits high rectification characteristics observed in our GaN NACDs, which can be attributed to three key factors. First, the utilization of the ALD technique for preparing the air channels has significantly improved their uniformity compared to those prepared using the PECVD process (refer to Section 2.1 and Figure S3, Supporting Information). This improvement reduces the surface roughness of the Au electrode, leading to a decrease in its field emission capability^[^ ^25^ ^]^ of the Au electrode from 10^−5^ to 10^−8^ A at 3 V. As a result, the rectification ratio of the device is elevated from 10^2^ to 10^5^. The simulation of the electric field distribution on high and low roughness surfaces is shown in Figure 2d, indicating a significant difference in the electric field on various roughness surfaces. Additional simulations of the dependence of field emission performance on roughness are shown in Figure S13 (Supporting Information). Second, the significant disparity in work function between Au and GaN (5.1 and 4.0 eV, respectively) induces a pronounced bending of the GaN‐air interface barrier at a given voltage, as shown in Figure 2e,f. Consequently, GaN NACDs exhibit a low *V*_ON_ and a large output current under positive bias. Lastly, the surface state of GaN also plays a crucial role in shaping the output characteristics of GaN NACDs. When a positive or negative bias is applied to the Au electrode, the electrons on the GaN surface undergo an accumulation or depletion state, respectively. Under positive bias, there is an augmented voltage drop across the air channel as compared to the depletion case due to the formation of a depletion region within the GaN. Consequently, this results in a more favorable electron emission from GaN when compared to Au under negative bias conditions.^[^ ^2^ ^]^

### 2.3 Dynamic Rectification Characteristics

Diodes play a pivotal role in various fields, such as energy conversion,^[^ ^39^ ^]^ communication systems,^[^ ^40^ ^]^ and integrated circuits,^[^ ^41^ ^]^ by facilitating the conversion of AC signals into DC signals. In practical applications, electronic devices often operate in complex electrical signal environments. Therefore, it is crucial to understand the dynamic rectification behavior of diodes under non‐steady‐state signals for optimizing circuit design and improving circuit efficiency and stability. In this paper, the dynamic rectification characteristics of NACDs were investigated by driving them with square wave and sine wave signals of different frequencies using a semiconductor parameter analyzer. This investigation endeavors to fill the gap in knowledge regarding the dynamic rectification properties of NACDs.

**Figure 3:**
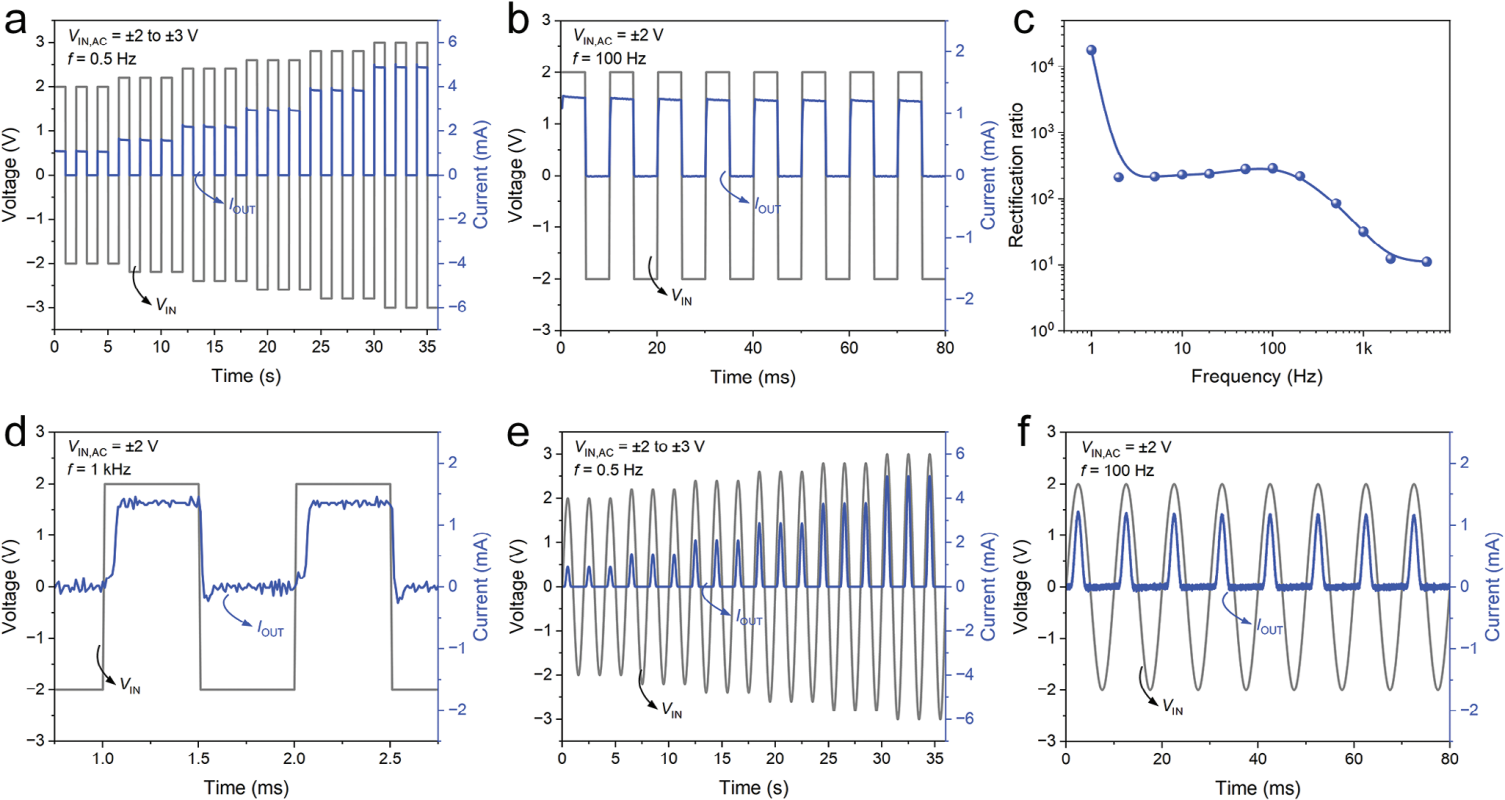
Dynamic rectification measurements of GaN NACD with square electrode. a) Rectified output currents with different square wave input voltages ranging from ± 2 to ± 3 V (the input signal frequency is 0.5 Hz). b) Output current with square wave *V*_IN,AC_ = ±2 V, 100 Hz. c) Rectification ratios for square wave input frequencies ranging from 1 Hz to 5 kHz (the input voltage is ± 2 V). d) Output current at square wave *V*_IN,AC_ = ±2 V, 1 kHz with an RC delay of ≈80 µs for both the ON‐ and OFF‐states. e) Rectified output currents with different sine wave input voltages ranging from ± 2 to ± 3 V (the input signal frequency is 0.5 Hz). f) Output current with sine wave *V*_IN,AC_ = ±2 V, 100 Hz.

**Figure** **3**a illustrates the rectification performance of the GaN NACD under square wave signals of varying voltage amplitudes. The blue curve represents the rectified output current (*I*_OUT_), while the grey curve represents the input voltage (*V*_IN_) square‐wave pulse signals. These pulse signals have a peak‐to‐peak range from ±2 V to ±3 V, with a step size of 0.2 V, a pulse frequency of 0.5 Hz, and a duty cycle of 50%. As the amplitude of the input square wave voltage increases, the device exhibits an increase in the output current and demonstrates stable rectification characteristics. In addition, we investigated the rectification characteristics of GaN NACD under square waves of different frequencies. The test data, with voltage amplitudes of ±2 V and frequencies ranging from 1 Hz to 5 kHz, are presented in Figure S14 (Supporting Information). Figure 3b shows the rectification characteristics for an input voltage with a pulse frequency of 100 Hz and a duty cycle of 50%. The dynamic rectification of square waves depicted in Figure 3b and Figure S14 (Supporting Information) shows that the rectification demonstrates a peak *I*_OUT_ of ≈1.2 mA at all frequencies. However, it is noteworthy that the rectification characteristics deteriorate as the frequency increases to 5 kHz, with the reverse current rising to 10^−4^ A.

To investigate the influence of square waves with different frequencies on the device's rectification characteristics, we compared the dynamic rectification ratio at various frequencies with a 2 V input, as shown in Figure 3c. The device exhibits an output current of 1.16 mA @ 2 V in the DC test, with a static rectification ratio of 6.46 × 10^4^ at 2 V, as shown in Figure 2a. Here, the dynamic rectification ratio is defined as the ratio of the average ON‐state current to the average OFF‐state current driven by a square wave. Upon surpassing a frequency of 1 Hz, the dynamic rectification ratio exhibits a sharp decline from 10^4^ to 10^2^ before gradually stabilizing. As the frequency is increased beyond 100 Hz, the dynamic rectification ratio continues to decline, dropping to 10 at a frequency of 5 kHz. This decrease in the dynamic rectification ratio with increasing input signal frequency can be attributed to the rise in OFF‐state current and current fluctuation. It is probably due to the parasitic capacitance^[^ ^42^ ^]^ introduced by the large overlap between the Au electrode and the GaN. Furthermore, at an input frequency of 1 kHz, the square wave rectification exhibits an RC delay of ≈80 µs for both the ON‐ and OFF‐states, as illustrated in Figure 3d. This delay can be further improved by optimizing the device structure and reducing the parasitic effects of the measurement setup.^[^ ^43^ ^]^

The dynamic rectification of the GaN NACD under sine signals was also investigated, as presented in Figure 3e,f. The research findings indicated that our device maintains stable and reproducible rectification characteristics when subjected to sine signals with varying voltage amplitudes and frequencies.

### 2.4 Neutron Irradiation Resistances

The increasing demand for radiation‐hardened electronics in high‐radiation environments, including space exploration, nuclear reactors, particle accelerators, and other high‐radiation environments, has highlighted the urgent need for technologies to withstand such conditions.^[^ ^44^ ^]^ Radiation‐induced damage can be generally classified into three types: single‐event effects (SEE), total ionizing dose (TID) effects, and displacement damage (DD).^[^ ^45^ ^]^ In CMOS devices, SEE occurs when high‐energy ions deposit charge in the depletion region, leading to device failure.^[^ ^4^ ^]^ However, SEE is not well‐defined in NACTs, which lack a depletion region in the electron transport channel. TID effects in silicon transistors result in the ionization of electrons and holes in the insulating region, leading to uneven charge distribution and potential breakdown of the insulating layer. In the case of NACDs, the channels are fully covered by the metal gate or anode, preventing direct contact between the channel and the insulating layer material. Consequently, the TID effect on NACDs is likely to be negligible.^[^ ^4^, ^46^ ^]^ DD effects occur when high‐energy particle radiation displaces atoms or generates defects in the lattice structure.^[^ ^47^ ^]^ If atoms acquire sufficient energy, they can be knocked out of their lattice positions and move to interstitial positions. This process could degrade the performance of NACTs by affecting the properties of cathodes and anodes.

**Figure 4:**
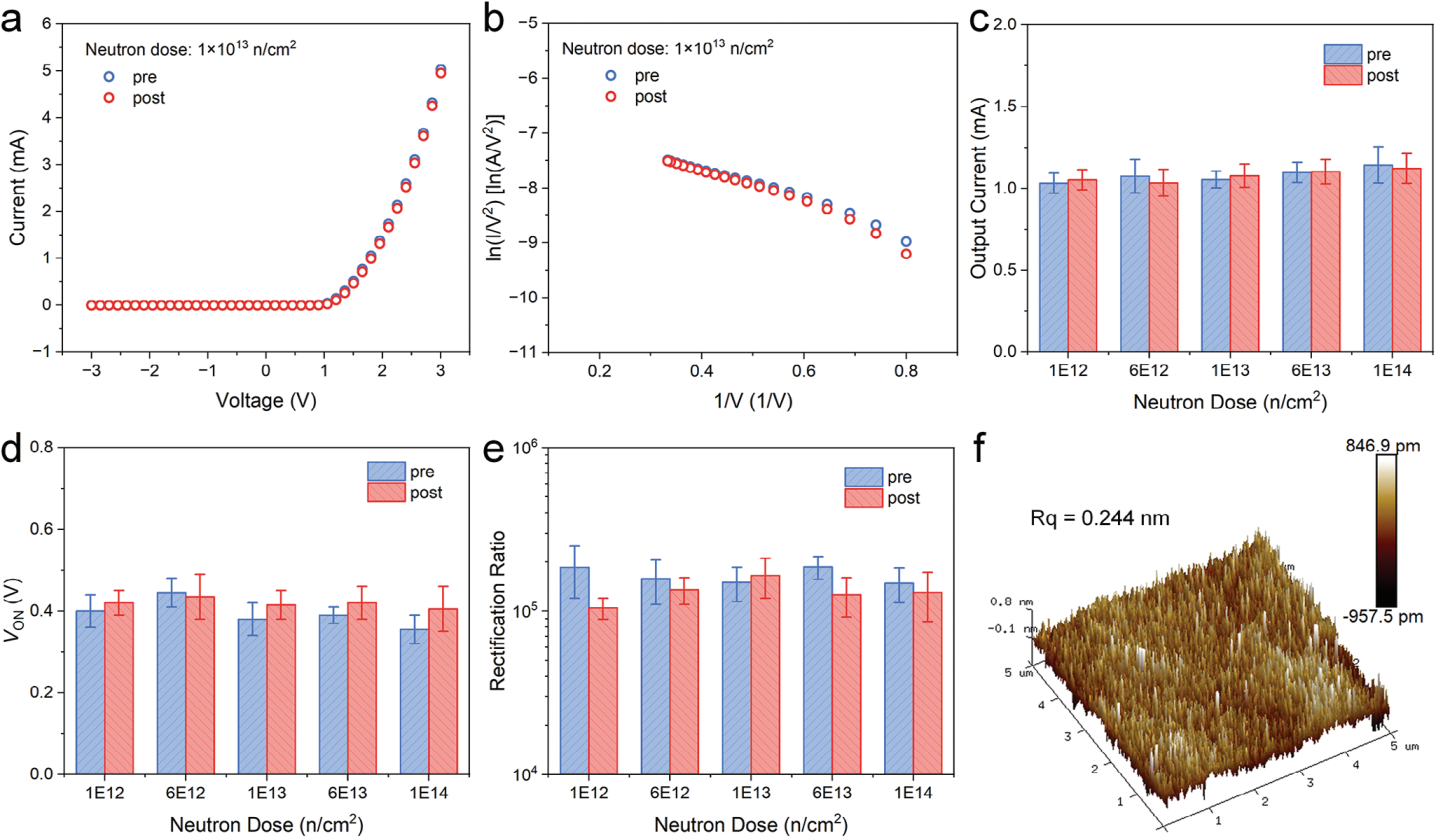
The neutron irradiation performance of GaN NACD with the square electrode. a) *I*–*V* characteristics of the device before and after neutron irradiation with a dose of 1 × 10^13^ n cm^−2^. b) Corresponding FN curves of the device before and after neutron irradiation. c,d,e) Output current (c), turn‐on voltage (d), and rectification ratio (e) at 2 V under different neutron irradiation doses. f) AFM image of the surface roughness of GaN NACD after neutron irradiation with a dose of 1 × 10^14^ n cm^−2^.

Research by Vanderbilt University^[^ ^48^ ^]^ and NASA^[^ ^49^ ^]^ on the radiation tolerance test of NACTs has provided valuable insights. Nevertheless, the radiation tolerance of GaN‐based NACDs still needs to be extensively studied. In response to this, we conducted displacement damage tests by exposing the devices to 1.2 MeV fast neutron radiation at varying doses of 1 × 10^12^, 6 × 10^12^, 1 × 10^13^, 6 × 10^13^, and 1 × 10^14^ n cm^−2^. The performance changes were assessed by testing five identical devices under each neutron dose. The pre‐and post‐irradiation output currents were measured and depicted in Figure S15 (Supporting Information) of Supplementary Information. As **Figure** **4**a illustrates, the emission current of the GaN NACDs remains consistent before and after neutron irradiation, demonstrating no significant current degradation. The corresponding FN curves also show no linear slope changes, as shown in Figure 4b, indicating that neutron radiation did not significantly affect the cathode work function and field enhancement factor. Figure 4c–e further demonstrates that varying doses of fast neutron irradiation did not result in significant shifts in the output current, *V*_ON_, and rectification ratio of GaN NACDs. In comparison, the threshold voltage of GaN power devices experienced a rise after 1 MeV fast neutron irradiation under the influence of 1 × 10^14^ n cm^−2^,^[^ ^50^ ^]^ while AlGaN‐based light emitting diodes displayed a reduction of >35% in output power under the same neutron radiation conduction.^[^ ^51^ ^]^ These findings suggest that GaN NACDs have a favorable radiation tolerance compared to other wide bandgap devices.

The resistance of GaN NACDs to neutron radiation is due to their unique structure and material properties. The “junction‐free” nature of NACDs makes them less susceptible to radiation damage than traditional semiconductors.^[^ ^9^ ^]^ High‐energy ion radiation typically requires a certain travel distance to induce atomic displacement and lattice defects.^[^ ^4^ ^]^ In the case of GaN NACDs, the nano air channels act as a transparent medium for fast neutron radiation, allowing most particles to pass the channels without significant energy loss. It is essential to consider the physical changes that neutron radiation may cause in the GaN cathode, such as swelling and displacement per atom (dpa) levels.^[^ ^52^ ^]^ However, the AFM observations in Figure 4f reveal no evidence of swelling or roughness changes on the GaN surface with a neutron irradiation dose of 1 × 10^14^ n cm^−2^. Research suggests moderate swelling in GaN typically requires radiation doses exceeding 1 × 10^15^ n cm^−2^.^[^ ^53^ ^]^ Furthermore, simulations by Zhang et al.^[^ ^54^ ^]^ using Geant4 software indicated that at a neutron energy of 1 MeV, the dpa of GaN material was only 1.01 × 10^−20^. This suggests that it is challenging for the 1 MeV fast neutron to displace GaN atoms from their lattice positions. Overall, the combination of the junction‐free design and the inherent properties of GaN contribute to the high radiation tolerance exhibited by GaN NACDs.

## 3 Conclusion

In summary, we demonstrated the high‐performance and rectifying properties of GaN NACDs with exceptional uniformity and precise control over nano air channels using IC‐compatible methods. Our achievement of nanometer‐scale precision in the length of the air channels, maintaining excellent uniformity and consistency with only a 1.5 nm difference from the target size, is noteworthy. The GaN NACDs exhibit a high rectification ratio and high performance at a turn‐on voltage of only 0.3 V, attributed to the reduced surface roughness of the Au electrode and significant work function difference between the cathode and anode. This study also highlights the potential of dynamic rectification in practical applications, validated through square wave and sine wave measurements at different voltages and frequencies. Furthermore, the junction‐free nature of GaN NACDs provides immunity to displacement damage caused by fast neutron radiation, making them highly suitable for use in radiation environments such as space and nuclear applications. Overall, our research provides valuable insights into the design of rectification characteristics in NACDs and offers hope for developing next‐generation low‐power, high‐performance, and radiation‐tolerant devices.

## 4 Experimental Section

### Device Fabrication

The fabrication of GaN NACDs involved a combination of IC‐compatible techniques. Initially, SiO_2_ layers with thicknesses of 50, 60, and 70 nm were deposited on a pre‐cleaned n‐GaN surface (Si: 3 × 10^18^ cm^−3^) using atomic layer deposition (ALD). Subsequently, photolithography was employed to define the electrode patterns, followed by electron beam evaporation to produce a 100 nm/10 nm thick Au/Ti electrode, wherein Ti served as the adherent layer. The circular electrode has a radius of 1273 µm and the square electrode has a side length of 2000 µm, so they have an equal circumference of 8000 µm. A lift‐off process was then employed to remove the metal over the photoresist. Next, the SiO_2_ outside the Au electrode was eliminated using RIE. In addition, an additional Au electrode was deposited using electron beam evaporation to serve as a contact electrode for GaN. Finally, a nano air channel was created by selectively removing a portion of the SiO_2_ between the Au edge and the GaN through 80 s of BOE wet etching. The height of the nano air channel is equal to the thickness of the oxide layer, and the depth is ≈400 nm.

### Device Cross‐Section Cutting

A FIB system (Helios G4 UX) was employed to cut the device to obtain the cross‐sectional profile. The Ga^+^ ions were accelerated at a voltage of 30 keV, and an ion beam current of 20 nA was employed for this procedure.

### Device Performance Measurement

The static current–voltage (*I*–*V*) characteristics of GaN NACDs were measured using a semiconductor parameter analyzer (FS‐Pro) under both ambient and vacuum conditions (3 × 10^−4^ Pa). In addition to measuring the *I‐* *V* characteristics, the semiconductor parameter analyzer was employed to drive the GaN NACDs and assess their dynamic rectification properties by applying AC voltage signals (square wave and sine signals at various frequencies) while recording the corresponding output current signals.

### Multiphysics Simulation

The commercial finite element method software (COMSOL Multiphysics 5.6) was used to simulate the effect of electrode geometry, various cathode work functions, and surface roughness of the cathode on field emission performance.

## Conflict of Interest

The authors declare no conflict of interest.

## Supporting information

Supporting Information

## Data Availability

The data that support the findings of this study are available from the corresponding author upon reasonable request.
