# Supplementary material for: GaN Nano Air Channel Diodes: Enabling High Rectification Ratio and Neutron Robust Radiation Operation
Source: Adv Sci (Weinh). 2024 Jun 27;11(33):2310300. doi: 10.1002/advs.202310300 (PMC11434220; doi:10.1002/advs.202310300)
Supplement: Supplementary file 1 — Supporting Information [file ADVS-11-2310300-s001.docx]

Supporting Information for

**GaN Nano Air Channel Diodes: Enabling High Rectification Ratio and Neutron Robust Radiation Operation**

*Yazhou Wei^1,2^, Feiliang Chen^1,2,3^, Yu Zhang^1,2^, Ruihan Huang^1,2^, Haiquan Zhao^1,2^, Mo Li^1,2,3,*^, and Jian Zhang^1,2,3^*

^1^School of Electronic Science and Engineering, University of Electronic Science and Technology of China, Chengdu 611731, China.

^2^Institute of Advanced Millimeter-Wave Technology, University of Electronic Science and Technology of China, Chengdu 611731, China.

^3^Yangtze Delta Region Institute, University of Electronic Science and Technology of China, Huzhou 313000, China.

*E-mail: limo@uestc.edu.cn.

**Figure S1:** SEM images of device cross-section before and after BOE wet etching.

**Figure S2:** SEM images and the corresponding binary visualizations of the 50 nm, 60 nm, and 70 nm nano air channels.

**Figure S3:** Size distribution of air channel prepared by PECVD process.

**Figure S4:** Surface roughness of GaN using AFM.

**Figure S5**: Schottky emission fit of *I*–*V* data of GaN NACDs with square and circular electrodes.

**Figure S6:** Simulation of the effect of electrode shape on electric field.

**Figure S7**: Cyclic *I*-*V* characteristics of GaN NACD with square electrode.

**Figure S8:** FN curves of square and circular electrode devices with Au as the cathode.

**Figure S9:** Electrical performance of devices with different air channel lengths.

**Figure S10**: Simulation study of material work function on device performance.

**Figure S11:** Breakdown voltage characteristics of a GaN NACD with a 50 nm air channel.

**Figure S12:** Stability and reliability measurements of GaN NACD.

**Figure S13:** Simulating the effect of cathode surface roughness on field emission performance.

**Figure S14:** Rectification characteristics of the device with input square wave signal frequencies from 1 Hz to 5 kHz.

**Figure S15:** Comparison of *I*-*V* characteristics of the device before and after neutron irradiation.

**
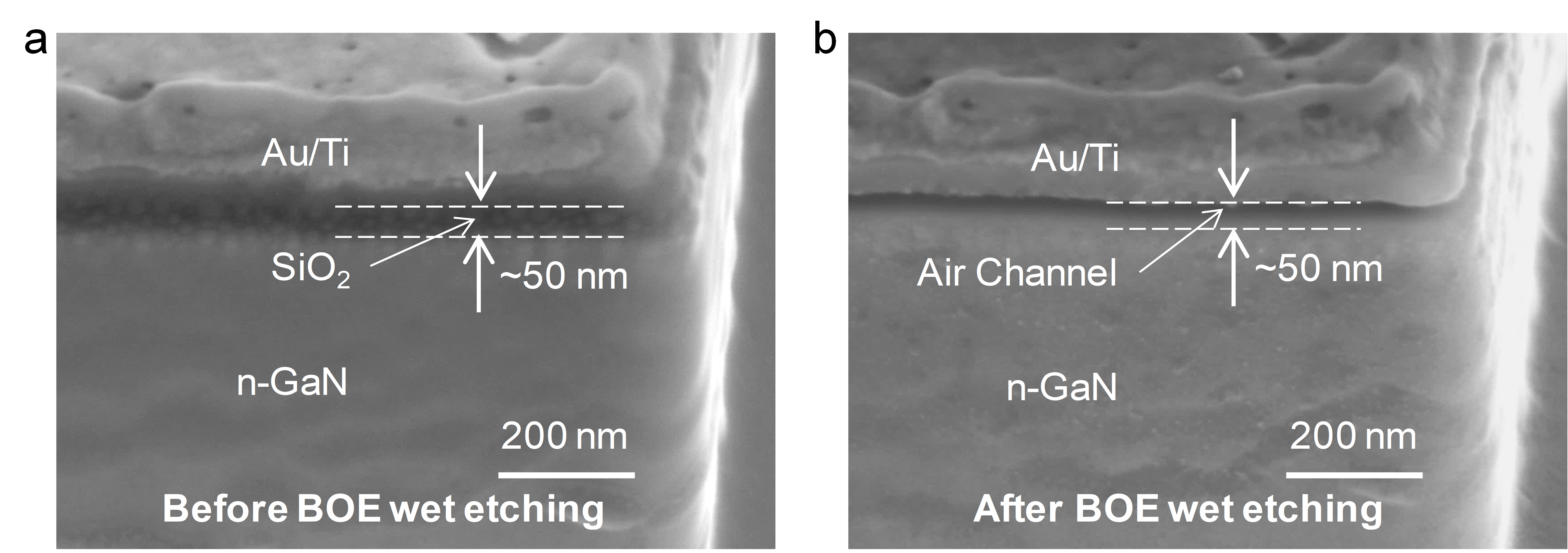
**

**Figure S1**. SEM images of the GaN NACD’s cross-section before (a) and after (b) BOE wet etching.

**
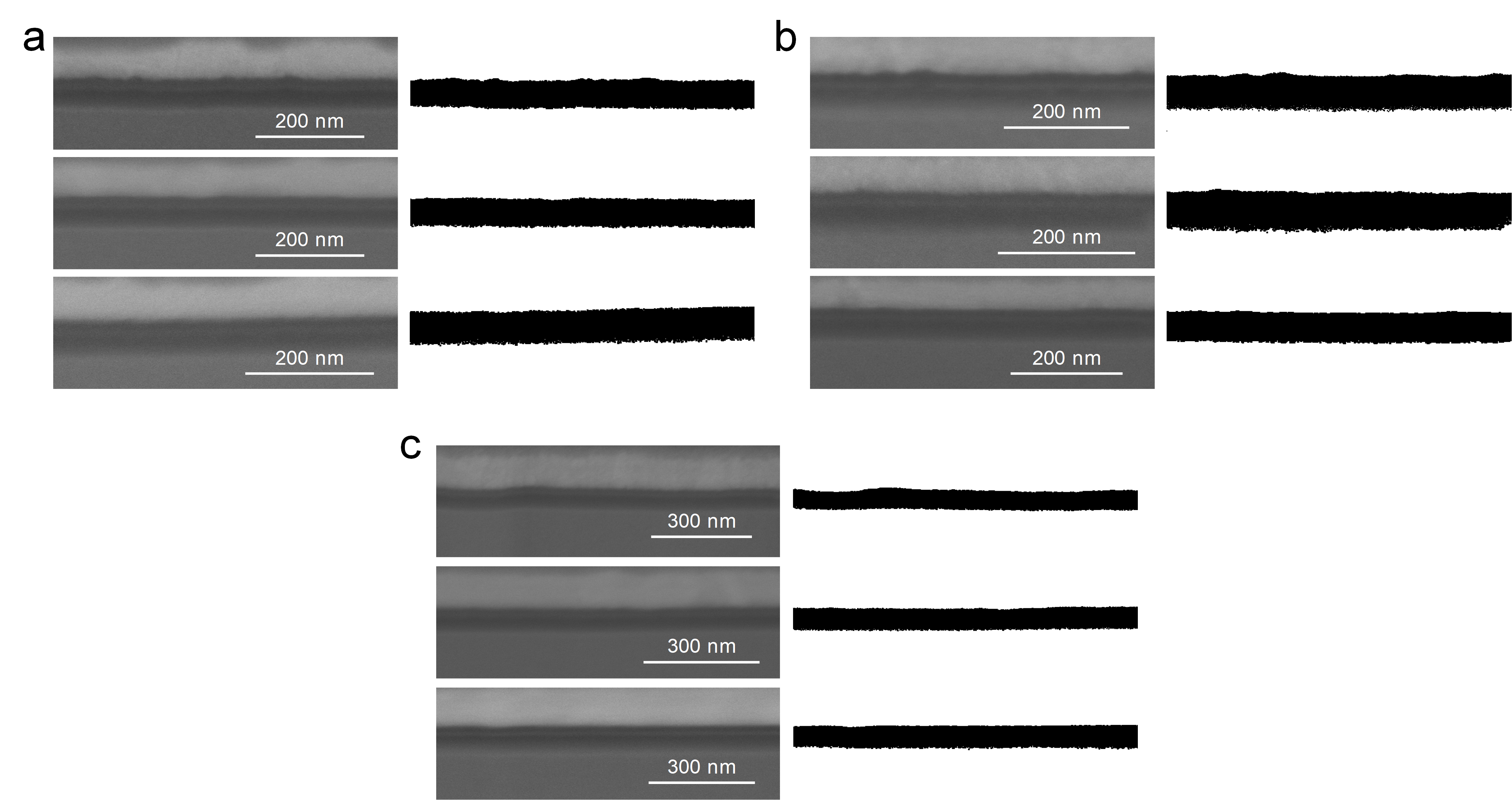
**

**Figure S2.** SEM images and the corresponding binary visualizations of the 50 nm (a), 60 nm (b), and 70 nm (c) nano air channels between Au and GaN, with only the regions within the air channels shown as dark pixels.


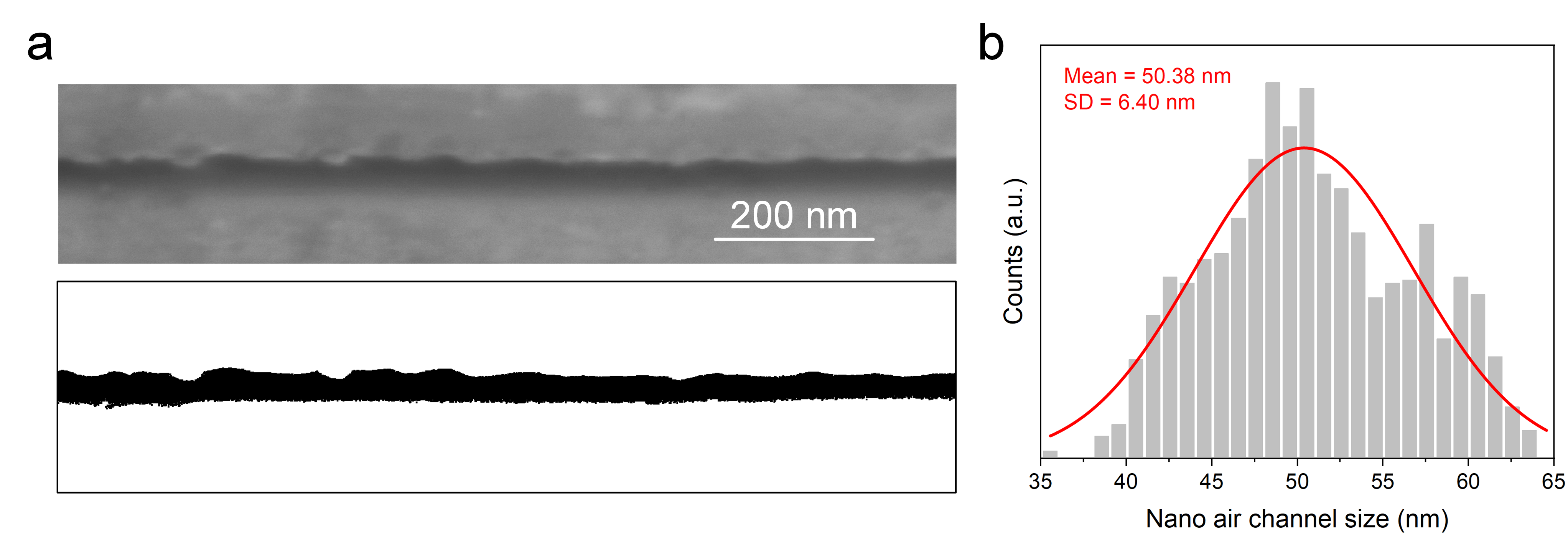


**Figure S3.** Size distribution of air channel prepared by PECVD process. a) SEM image and corresponding binary visualization of the nano air channel, where only the areas within the air channel are displayed as dark pixels. b) The histogram summarizing the size distribution of 50 nm. The majority peak is centered around 50.38 nm for the air channel dimensions, with standard deviations of 6.40 nm.


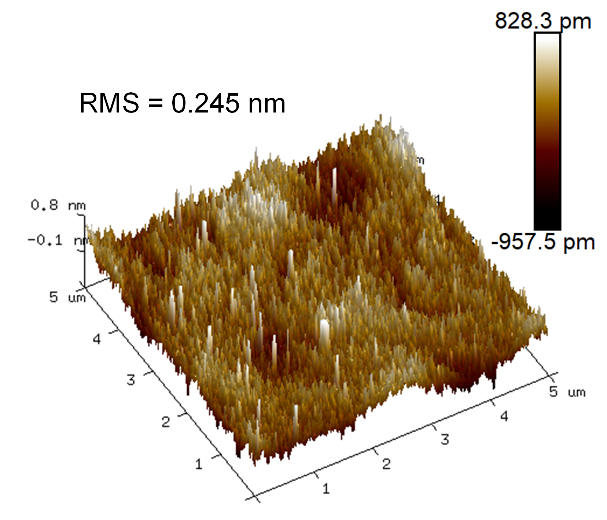


**Figure S4**. Surface roughness of the GaN using AFM.


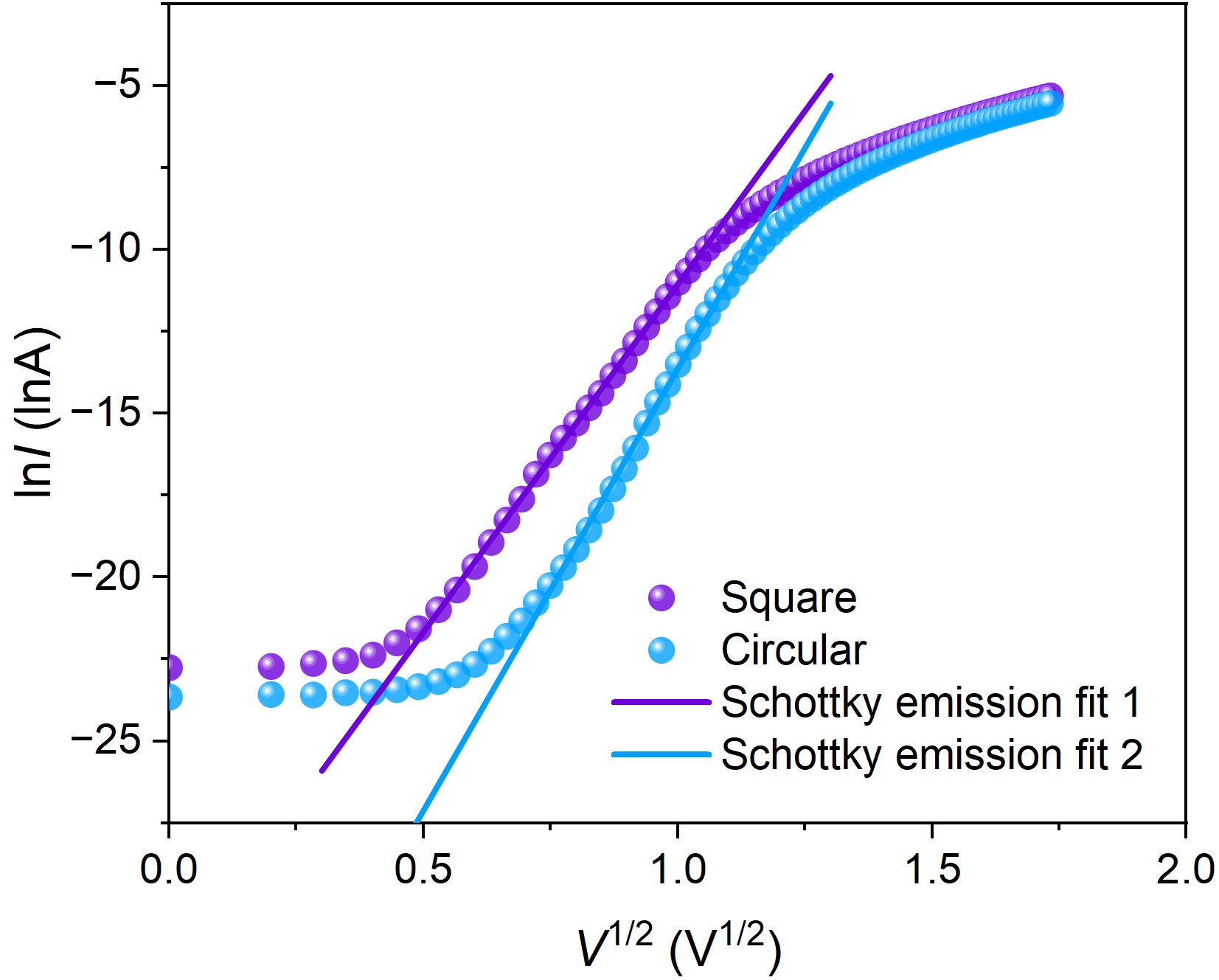


**Figure S5**. Schottky emission fit of *I*–*V* data of GaN NACDs with square and circular electrodes in Richardson–Schottky coordinates (ln*I* versus *V*^1/2^).


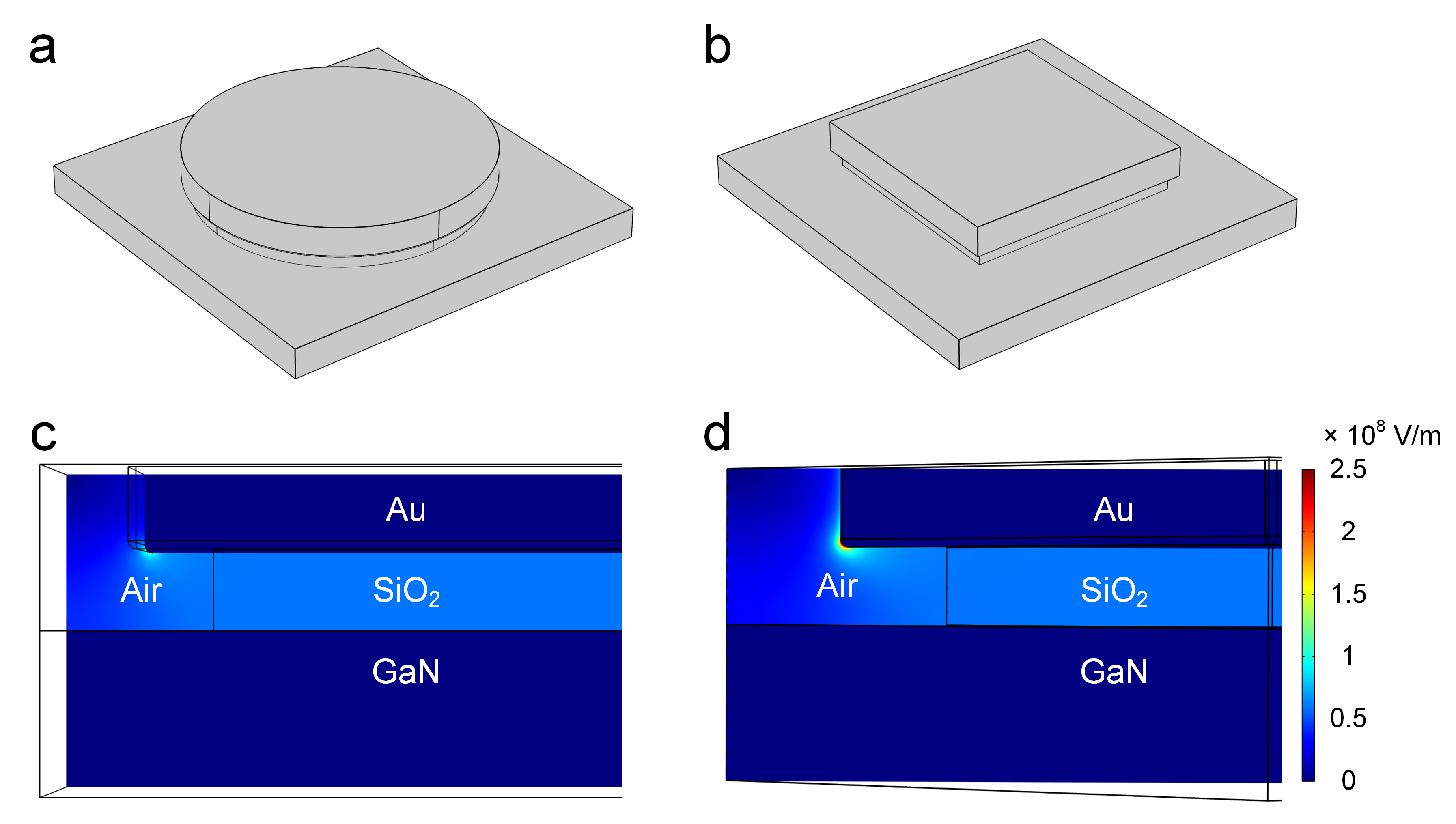


**Figure S6**. Simulation of the effect of electrode geometry on electric field. a,b) COMSOL models with circular (a) and square (b) electrodes with the same electrode circumference. The channel length is set to 50 nm and the anode voltage is 3 V in the simulation. c,d) Electric field distributions in cross sections with circular (c) and square (d) electrode. The maximum electric fields at the edges of the circular and square electrodes are 1.2 × 10^8^ V/m and 2.4 × 10^8^ V/m, respectively.


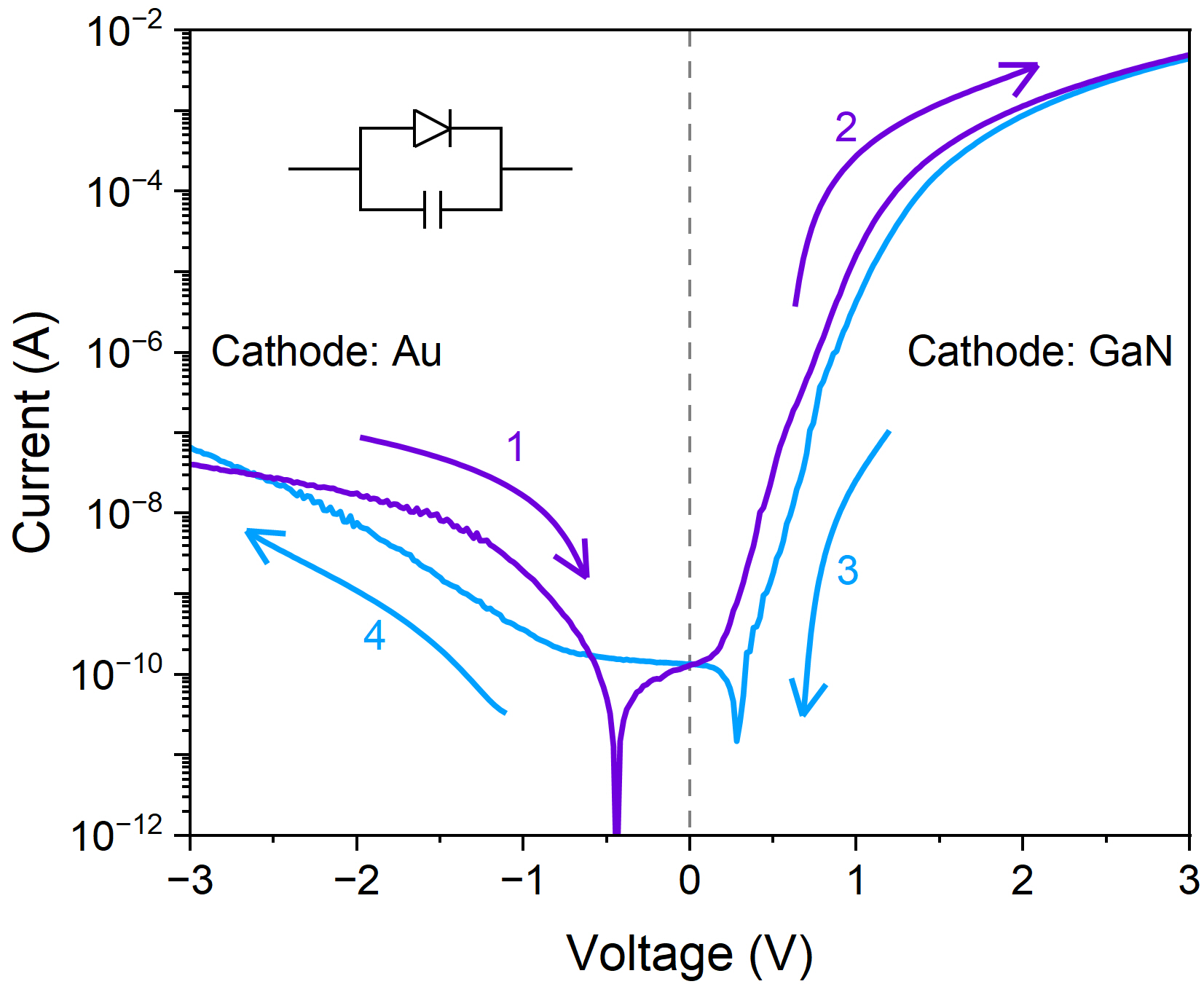


**Figure S7**. Cyclic I-V characteristics of GaN NACD with square electrode.


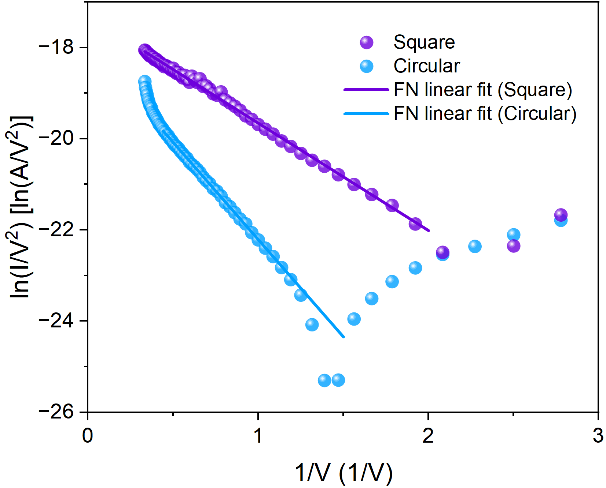


**Figure S8**. FN curves of devices with square and circular electrodes with Au as the cathodes.

**
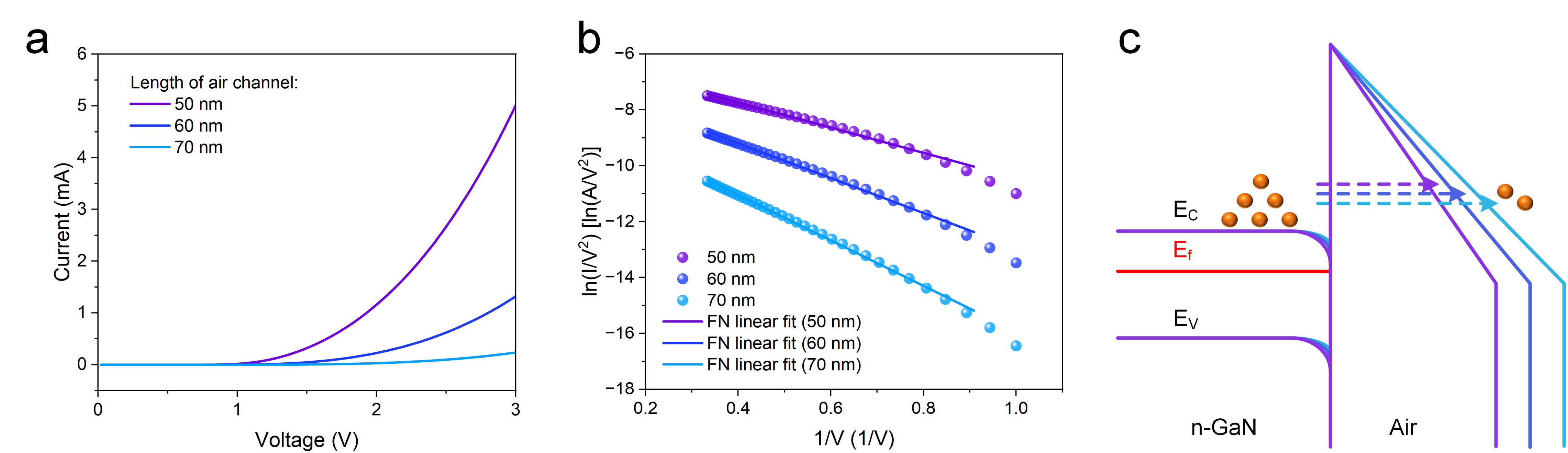
**

**Figure S9.** a) I-V characteristic curve of devices with different air channel lengths (50 nm, 60 nm, and 70 nm) under positive bias. At a bias voltage of 3 V, the output currents of the three devices are 5.02 mA, 1.32 mA, and 0.24 mA, respectively. b) FN curves of GaN NACDs with different air channel lengths. c) Schematic diagram of energy bands. E_C_, E_F_, and E_V_ denote the conduction band, Fermi level, and valence band, respectively.


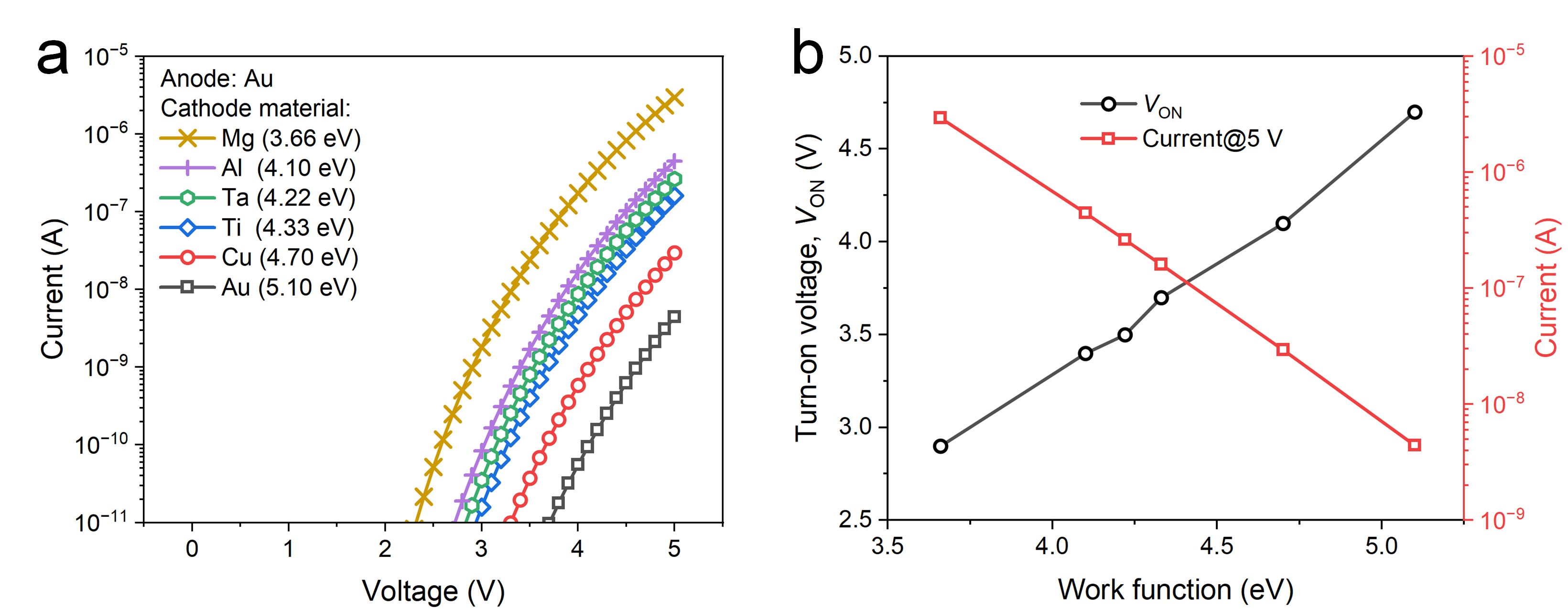


**Figure S10**. Simulation of the performance of NACDs with various cathode work functions. a) *I*-*V* characteristics of NACDs with various cathode work function values. The air channel length is set to 50 nm and the field enhancement factor is 100. b) Corresponding turn-on voltages and output currents at 5 V.


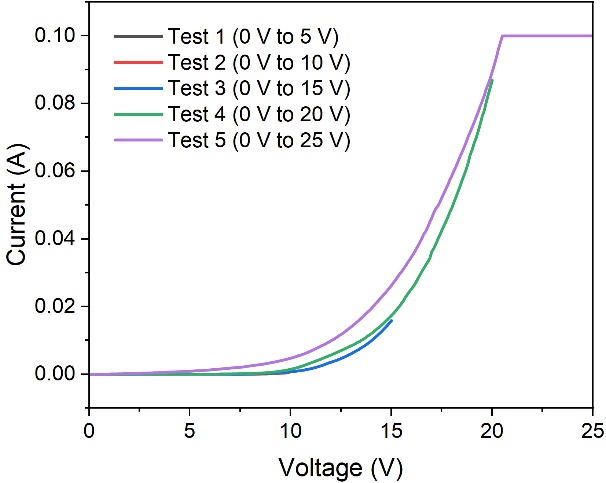


**Figure S11.** Breakdown voltage characteristics of the GaN NACD with a 50 nm air channel, which has a breakdown voltage of above 25 V.


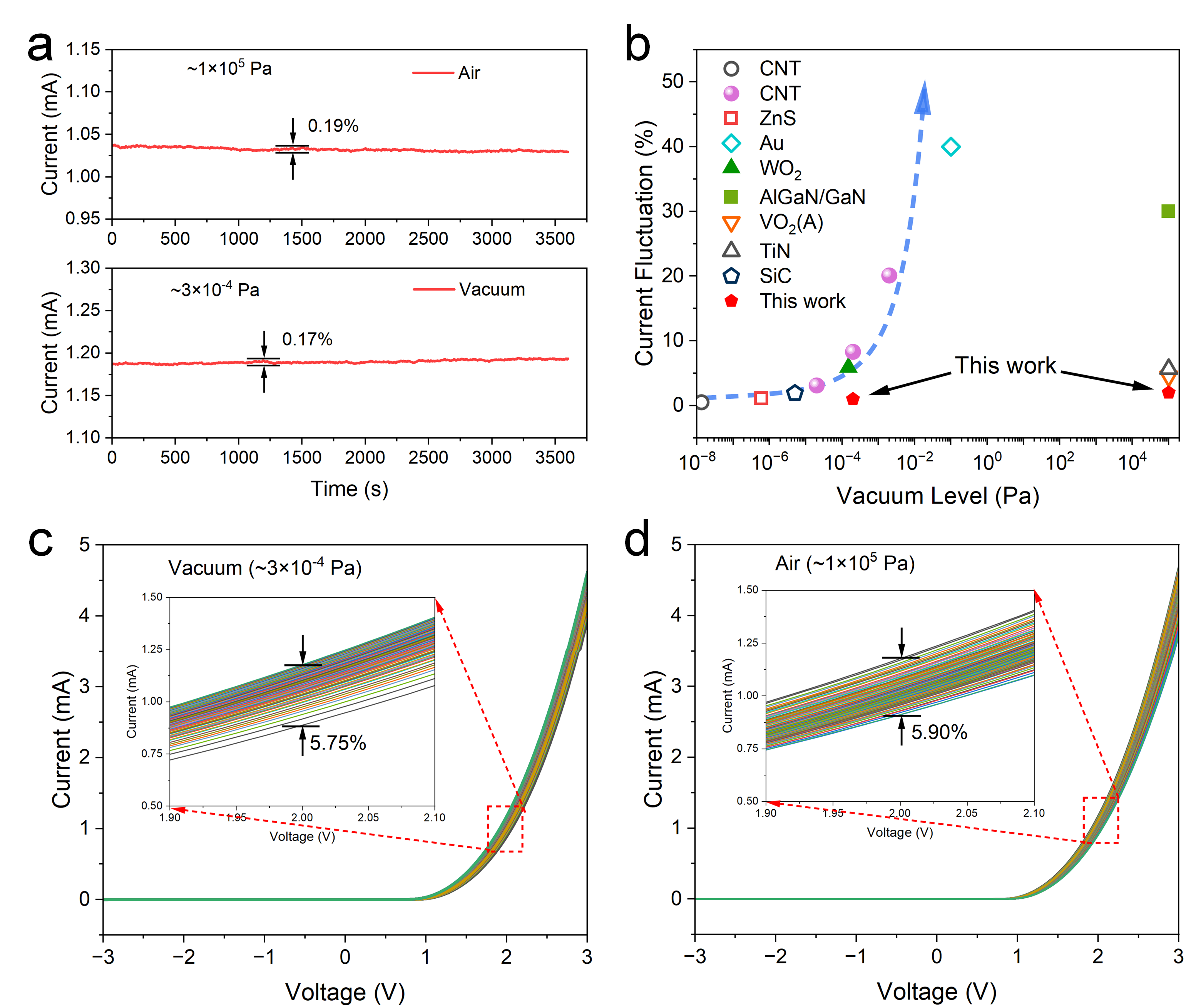


**Figure S12**. Stability and reliability measurements of GaN NACD. a) The output current curve of the short-term stability test of the GaN NACD. The device exhibits low output current variations, with relative standard deviations (RSD) of 0.19% and 0.17% for the output currents and 0.71% and 0.87% for the current variations over 60 minutes in vacuum and atmospheric conditions, respectively. b) Comparison of stability data with published work^[1-8]^. c) The output curve of the device repeated 100 times under vacuum conditions, with an RSD of the output current extracted at 2 V calculated to be ±5.7%. d) The output curve of the device repeated 100 times under normal pressure conditions, with an RSD of 5.90% for the output current.


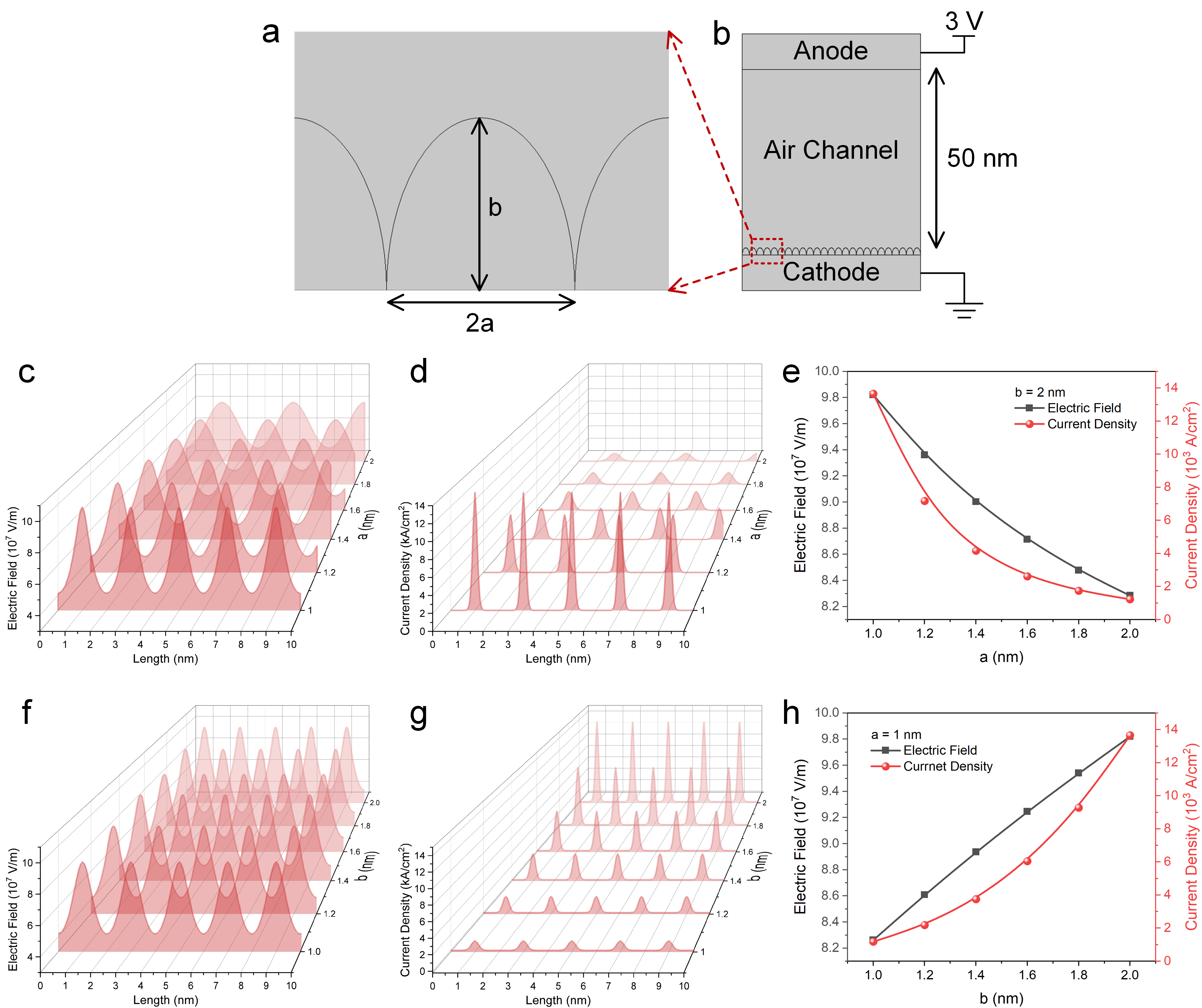


**Figure S13**. Simulating the effect of surface roughness of cathode on field emission performance. a) Schematic diagram of a semi-elliptical unit structure on a rough surface, with a horizontal axis of a and a vertical axis of b. b) The structural diagram of the roughness model shows a length of 50 nm between the anode and the roughness surface, with the anode biased at 3 V, while the cathode is grounded. c,d) The electric field (c) and current density distribution (d) simulated on a cathode with a length of 10 nm under different horizontal axis a value and a constant value of 2 nm for b. e) The maximum electric field and current density values on the semi elliptical surface of the roughness element under different horizontal axis a values. f,g) The electric field (f) and current density distribution (g) simulated on a cathode with a length of 10 nm under different vertical axis b values and a constant value of 1 nm for a. h) The maximum electric field and current density values on the semi-elliptical surface of the roughness element under different vertical axis b values.


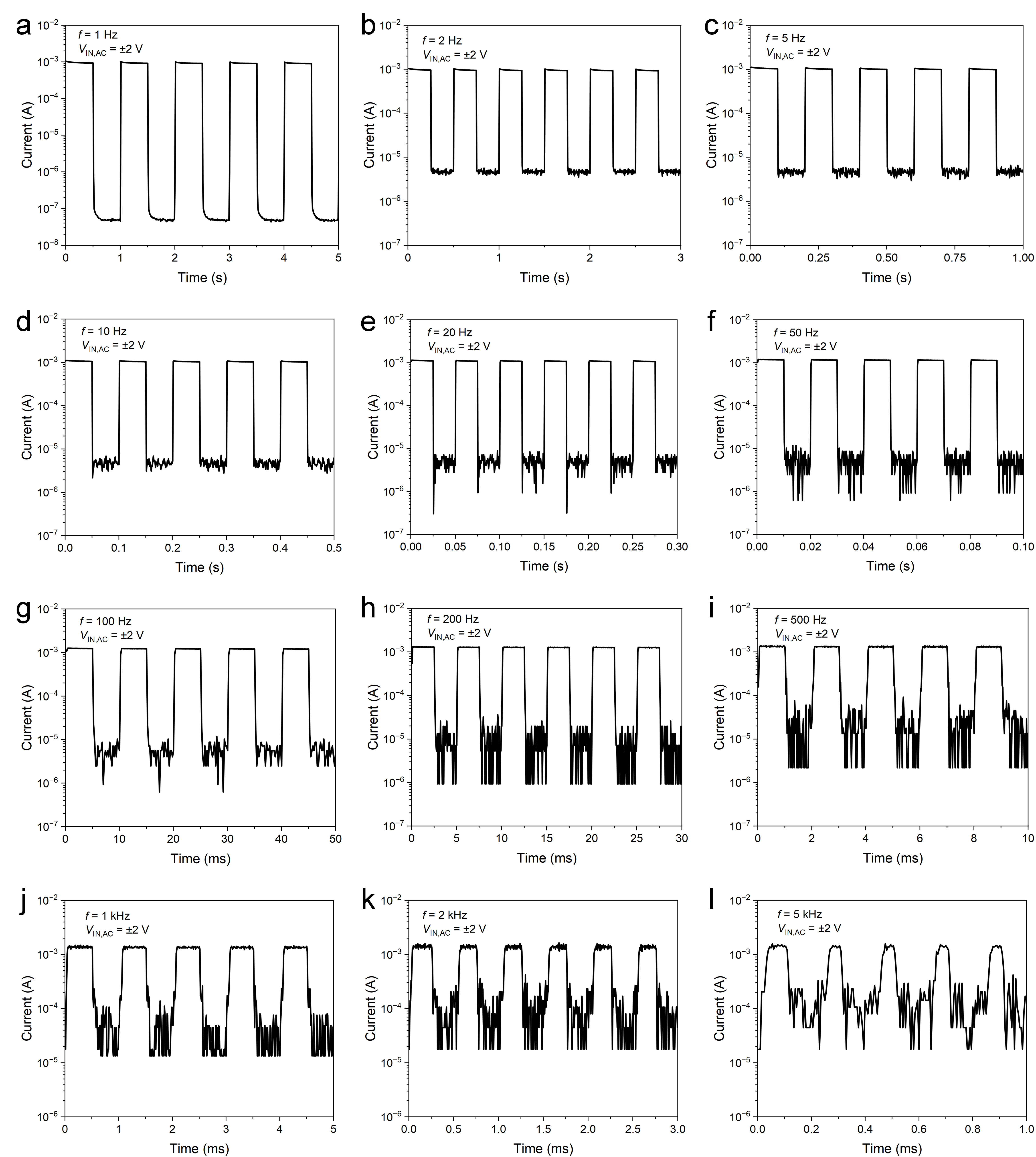


**Figure S14.** Rectification characteristics of the device with input square wave signal frequencies from 1 Hz to 5 kHz.


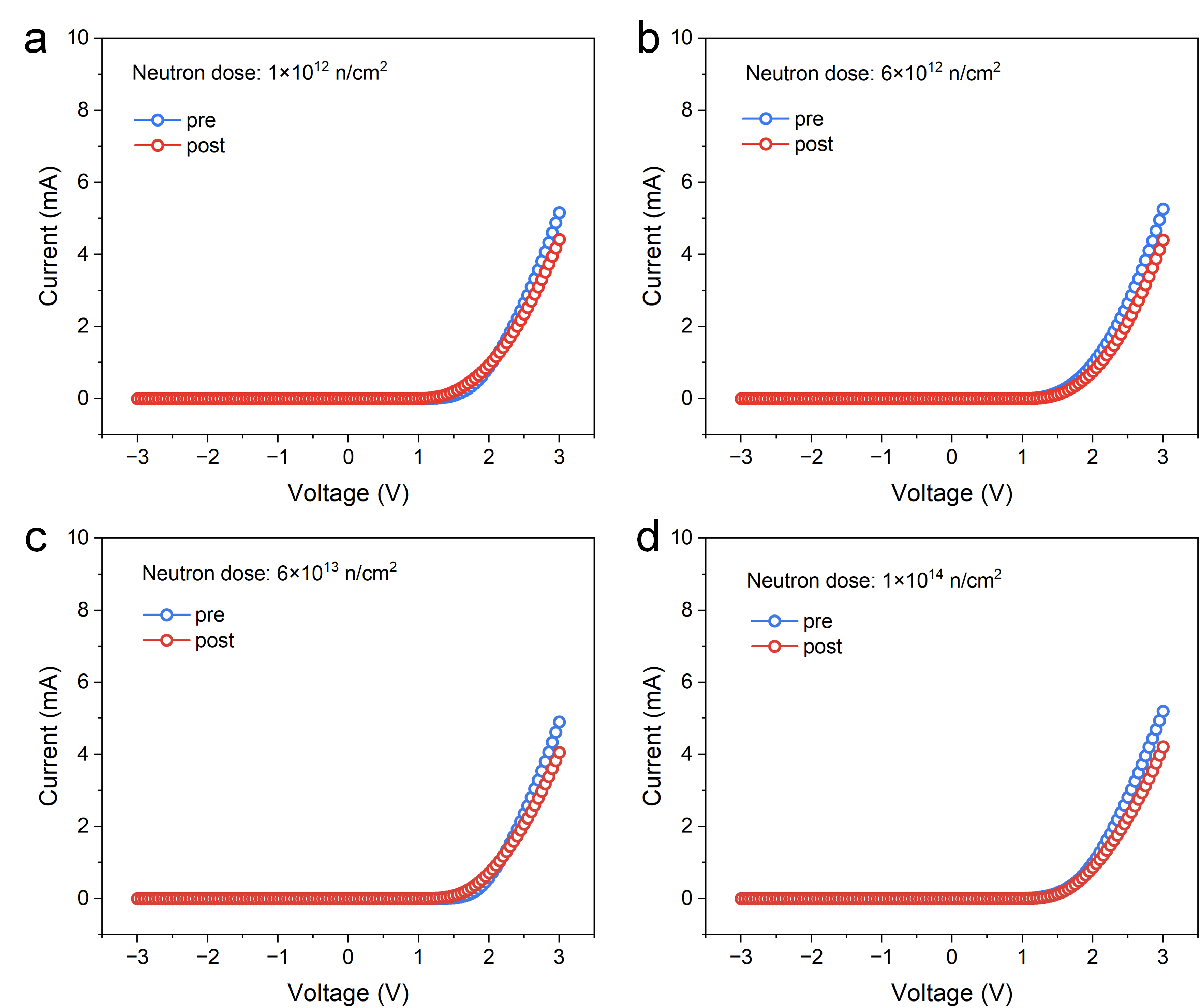


**Figure S15.** Comparison of *I*-*V* characteristics of the device before and after neutron irradiation with a dose of 1 × 10^12^ (a), 6 × 10^12^ (b), 6 × 10^13^ (c), and 1 × 10^14^ n/cm^2^ (d).

**References**

[1] Z. G. Chen, L. N. Cheng, H. Y. Xu, J. Z. Liu, J. Zou, T. Sekiguchi, G. Q. Lu, H. M. Cheng, *Advanced Materials* **2010**, 22, 2376.

[2] J. Xu, Q. L. Wang, Z. Tao, Y. S. Zhai, G. D. A. Chen, Z. Y. Qi, X. B. Zhang, *Ieee Transactions on Electron Devices* **2017**, 64, 2364.

[3] Y. Tian, X. Yang, T. Y. Guo, L. X. Peng, H. B. Gan, N. S. Xu, H. J. Chen, J. Chen, F. Liu, S. Z. Deng, *Advanced Materials Technologies* **2017**, 2, 1700029.

[4] M. Liu, W. B. Fu, Y. Yang, T. Li, Y. L. Wang, *Applied Physics Letters* **2018**, 112, 093104.

[5] D. S. Zhao, R. Liu, K. Fu, G. H. Yu, Y. Cai, H. J. Huang, Y. Q. Wang, R. G. Sun, B. S. Zhang, *Chinese Physics Letters* **2018**, 35, 038103, 038103.

[6] K. N. Yun, S. H. Lee, J. S. Han, Y. H. Song, C. J. Lee, *Nanotechnology* **2018**, 29, 085203.

[7] A. Nardi, M. Turchetti, W. A. Britton, Y. Chen, Y. Yang, L. Dal Negro, K. K. Berggren, P. D. Keathley, *Nanotechnology* **2021**, 32, 315208.

[8] J. F. Xiao, J. Z. Zhao, G. J. Liu, M. T. Cole, S. H. Zhou, K. Chen, X. C. Liu, Z. J. Li, C. Li, Q. Dai, *Nanomaterials* **2021**, 11, 3025.
